# Putative Abscopal Effect in Three Patients Treated by Combined Radiotherapy and Modulated Electrohyperthermia

**DOI:** 10.3389/fonc.2020.00254

**Published:** 2020-03-10

**Authors:** Mau-Shin Chi, Minesh P. Mehta, Kai-Lin Yang, Hung-Chih Lai, Ying-Chu Lin, Hui-Ling Ko, Yu-Shan Wang, Kuang-Wen Liao, Kwan-Hwa Chi

**Affiliations:** ^1^Department of Radiation Therapy and Oncology, Shin Kong Wu Ho-Su Memorial Hospital, Taipei, Taiwan; ^2^Ph.D. Degree Program of Biomedical Science and Engineering, National Chiao-Tung University, Hsinchu, Taiwan; ^3^Department of Radiation Oncology, Miami Cancer Institute, Miami, FL, United States; ^4^School of Medicine, Fu Jen Catholic University, New Taipei City, Taiwan; ^5^Department of Hematology and Oncology, Shin Kong Wu Ho-Su Memorial Hospital, Taipei, Taiwan; ^6^Institute of Molecular Medicine and Bioengineering, National Chiao-Tung University, Hsinchu, Taiwan; ^7^School of Medicine, National Yang-Ming University, Taipei, Taiwan

**Keywords:** modulated electrohyperthermia, immunotherapy, radiotherapy, abscopal effect, immune-related adverse events

## Abstract

**Purpose:** True abscopal responses from radiation therapy are extremely rare; the combination of immune checkpoint inhibitors with radiation therapy has led to more reports of the abscopal effect, but even in this setting, the genuine magnitude remains unknown and is still considered generally uncommon. We report the occurrence of what appears to be putative, durable abscopal tumor responses with associated auto-immune systemic reactions resulting from the combination of local radiotherapy (RT) and modulated electrohyperthermia (mEHT).

**Materials and Methods:** Data from advanced cancer patients treated palliatively with RT and mEHT between January and December 2017 were collected as part of a post-marketing safety monitoring program of mEHT therapy. We specified a minimum RT dose of 30 Gy and at least four mEHT treatments for reporting toxicities, which was the primary aim of the larger study.

**Results:** Thirty-three patients treated with RT and mEHT, both applied to the same lesion, were included. The median RT dose was 45.5 Gy in 20 fractions (fxs) and the median number of mEHT treatments was 12 (range, 4–20). Most patients had subsequent systemic therapy after one course of RT and mEHT. Three patients (9.1%) developed autoimmune toxicities. Case number 1 received RT and mEHT only; case number 2 had two cycles of concurrent low dose chemotherapy during RT; and case number 3 received concurrent immune checkpoint inhibitors. None of the three patients received any further systemic treatment due to obvious treatment-related autoimmune reactions which occurred rapidly after RT; one had autoimmune hepatitis, one had dermatitis herpetiformis and the third developed severe myasthenia gravis. Interestingly, what we surmise to be long-lasting abscopal responses outside the irradiated area, were noted in all three patients.

**Conclusion:** RT combined with mEHT could putatively result in enhancing immune responsiveness. These preliminary observational findings lead to the generation of a hypothesis that this combination induces both an *in-situ*, tumor-specific immune reaction and an anti-self-autoimmune reaction, in at least a small proportion of patients, and of those who experience the auto-immune response, tumor response is a concomitant finding. Mechanisms underlying this phenomenon need to be investigated further.

## Introduction

Local hyperthermia (HT) has long been regarded as an effective radio-sensitizer (1). Modulated electrohyperthermia (mEHT) therapy is one form of hyperthermia (2). mEHT utilizes the biophysical differences between malignant and normal cells for cancer-cell specific selective energy deposition, believed to be due to the lower impedance on the transmembrane protein clusters of malignant cells (3). The modulated electromagnetic frequency spectrum of 13.56 MHz from mEHT is similar to the alternating electrical field generated from tumor treating field therapy (TTField, Novocure, Inc.) (4–6). mEHT applies lower power than conventional HT, thus interstitially measured average temperature is relatively low, around 39.5°C. However, the corresponding transmembrane temperature differential across a cell is often quiet high (7). This transmembrane thermal stress destabilizes cell membranes, resulting in necrosis, and also enhanced apoptosis (8–11). This effect has been shown to enhance the release of heat shock proteins (HSPs), produce damage-associated molecular patterns (DAMP) and leads to increased immunogenicity, thereby mediating immunogenic cell-death (12). The electric field effect has also been demonstrated to activate intensive lymphocytic and dendritic cell penetration into tumor (13).

The abscopal effect from radiotherapy (RT) has been known for a long time and was interpreted as an immune-mediated effect (14). Despite millions of patients having been treated with RT, only 46 abscopal cases induced by radiation treatment alone have been described between 1969 and 2014 (15). In preclinical models, the combination of immune check point (ICP) inhibitors with RT has demonstrated abscopal effects, but human reports still remain sparse, largely restricted to melanoma and non-small cell lung cancer (16–21). However, in all such reports, it remains difficult to ascribe the abscopal effect purely to RT alone or in combination with immune enhancing therapeutics (22). A prospective trial of RT, with a granulocyte-macrophage colony stimulating factor in metastatic diseases, reported a surprisingly high abscopal effect of 27.6% (23). RT creates tumor and normal tissue damage, lysis, and antigen release for sustained *in-vivo* vaccination events. Thymus-derived regulatory T (Treg) cells played a critical role in the control of immune tolerance to self-antigens, however, they also resulted in reduced anti-tumor immunity (24). There were very few literature reports on how therapy related autoimmunity-mediated antitumor activity (25, 26).

We speculated that the incidence of the abscopal effect may be higher in patients who develop autoimmunity. Bakacs et al. reported that immune related adverse events (irAEs) induced by ipilimumab are very similar to the chronic graft vs. host disease that ensues allogenic bone marrow transplantation (27). Autoreactive T cells may bypass the negative selection pressure in the microenvironment of the tumor and differentiate to memory T cells that recognize both “self” and “tumor.” We report, we believe for first time, that patients treated with RT and mEHT may have a long treatment-free period once they unleash an autoimmune reaction, and further, that in such patients, successful salvaging through low-dose ICP inhibitors may be possible at tumor recurrence.

## Materials and Methods

We performed a single institution, observational case-cohort study for patients with metastatic cancers of various origins, treated with a combination of RT and mEHT, with a minimum RT dose of 30 Gy and at least four mEHT treatments, to report unexpected adverse events. This retrospective analysis was conducted as part of a post-marketing safety surveillance program after the approval of the mEHT device in the class III medical category in Taiwan. The study was approved by the Institutional Review Board and was conducted according to the guidelines of Good Clinical Practice.

### Patient Selection

Enrolled patients were 20 years of age or older, presented with inoperable, recurrent, or metastatic diseases, requiring palliation with RT. In our study, all patients underwent concurrent RT and mEHT with or without systemic therapies, based on the underlying clinical condition. All institution-specific consent requirements were adhered to; written informed consent was obtained from the participants for the publication of the case series.

### Radiotherapy

RT was performed using conventional fractionation (and not hypofractionated) schedules, with a dose of 2 to 3.5 Gy per fraction (fx), five times per week to at least 30 Gy, as clinically appropriate and necessary. The clinical target volume (CTV) was defined as the gross tumor volume (GTV) plus a margin of 3–5 mm, based on the specific tumor type being addressed. Patients were treated with Elekta Synergy® (Elekta, Stockholm, Sweden) or TomoTherapy® (Accuray, Sunnyvale, CA, USA) with standard immobilization devices, using image-guided, modulated arc therapy with 6-MV photons for most of the patients. For patients who had received RT prior to the study, the original treatment plans were retrieved in every case of suspected overlap with the prior RT fields, and appropriate organ-at-risk constraints were adhered to.

### Hyperthermia (mEHT)

The mEHT treatment was applied using an EHY 2000+ hyperthermia device (OncoTherm GmbH, Germany). Treatment lasted for 60 min and was administered once weekly. A 30 cm in diameter circular electrode was placed at the irradiated tumor site, approximating placement at the radiation field isocenter. A 13.56 MHz radiofrequency (RF) was used with a real-time, automatic tuning device resulting in energy-transfer matching and ensuring a standard wave ratio of ~1 (the most ideal value). The power was initially set to 80 Watts (W) and a step-up protocol was applied to increase by 20–30 W every 5 min, until 150 W was reached for the remaining treatment duration. The goal for the target energy delivered was minimally set at 500 kJ per treatment. All appropriate vital sign monitoring during and after treatment was conducted as per standard practice. With this technique, intratumoral temperature measurement is typically not performed because the temperature elevation measured by a conventional thermocouple is usually <2°C (28). Adverse events were assessed throughout each treatment, which included heat sensitivity, skin burning, pain, and gastric discomfort.

### Outcomes Evaluation

The primary endpoint was toxicity, which was evaluated weekly and recorded using the Common Terminology Criteria for Adverse Events (CTCAE) version 4.0. during the RT and mEHT period and 2 months after. The secondary outcome was the radiologic response, which was evaluated on the irradiated lesions according to the Response Evaluation Criteria in Solid Tumors (RECIST) version 1.1 (29) every 3 months with CT, PET-CT, or tumor markers, based on a baseline selection diagnostic/imaging finding. The response categories of interest included complete response (CR), partial response (PR), stable disease (SD), and progressive disease (PD). Most patients received some kind of systemic treatment afterward. The length of follow-up was defined from the last day of RT to the last follow-up visit. Baseline measurements and changes in the neutrophil to lymphocyte ratio (N/L) before and after treatment were collected.

### Statistical Analysis

The impact of patient-, tumor-, and treatment-related factors on response was evaluated using a univariate and multivariable analysis. Survival curves were estimated using the Kaplan-Meier method. Fisher's exact test (two-tailed test) was used for evaluating 2 × 2 tables for significance. Statistical analyses were performed with the SAS statistical software (version 9.2; SAS Institute, Cary, NC, USA). *P* < 0.05 were set for statistical significance.

## Results

### Patient and Disease Characteristics

Thirty-three patients with recurrent or metastatic cancer was enrolled between January 2017 and December 2017. Patient characteristics are listed in Table 1. The median patient age was 59.3 years (range, 38–84 years). Breast cancer, lung cancer, hepatoma, cholangiocarcinoma, and urothelial carcinoma were the five most common disease entities. The thorax was the most commonly treated site (12 patients in total, including four lung cancer patients and eight for breast cancer) followed by abdomen (nine patients) and liver (six patients). During the RT and mEHT treatment, 16 patients received concurrent chemotherapy, nine had ICP inhibitors and six were treated with both agents. The median follow-up time was 11.6 months (range, 4–22.7 months) with no patients lost for follow-up.

**Table 1:** Patient characteristics.

**Characteristics**	**No**.	**%**
**Sex**		
Female	17	51.5
Male	16	48.5
Age, median, range, years	59.4	38–84 years-old
**Disease entities**		
Breast cancer	8	24.4
Lung cancer	4	12.1
Hepatocellular carcinoma	3	9
Cholangiocarcinoma	3	9
Urothelial carcinoma	3	9
Others	12	36.5
**Treatment before RT+ mEHT**		
Surgery	12	36.3
RT	19	57.6
ChT (include hormone, target therapy)	21	63.6
IO	0	0
Cht+IO	2	6
**Treatment during RT+ mEHT**		
ChT (include hormone, target therapy)	16	48.5
IO	9	27.2
ChT+IO	6	18.2
Treatment after RT+ mEHT		
Surgery	1	4.6
ChT (include hormone, target therapy)	13	59.1
IO	3	13.6
ChT+IO	5	22.7

*Cht, chemotherapy; IO, immune-Oncology*.

### Treatment

The median RT dose was 45.5 Gy (range, 30–66 Gy), and the mean GTV was 138.9 cm^3^ (ranged between 20 and 5064.7 cm^3^). The median number of mEHT treatment fractions was 12 (range, 4–20).

### Treatment Outcome and Toxicities

The combination of RT and mEHT treatment was well-tolerated. A full listing of adverse events during the RT plus mEHT treatment is provided in Table 2. Common treatment-related adverse events were grade 1 skin, and grade 2 myelotoxicities. Transient core body temperature elevation (>38°C), which resolved shortly after mEHT treatment, was noted in six patients; two obese patients had localized subcutaneous fat induration that persisted for several weeks, and then resolved. The most important adverse events that went beyond our expectation were autoimmune related toxicities (three out of 33 patients, 9.1%). One patient treated only with RT and mEHT developed grade 3 autoimmune hepatitis. One patient who was simultaneously treated with low-dose ICP (Yervoy® and Opdivo®) developed grade 3 myasthenia gravis, and another patient developed grade 2 autoimmune-related skin toxicity (dermatitis herpetiformis). All three patients who developed autoimmune toxicities had long lasting abscopal effects in the absence of any further subsequent systemic treatment.

**Table 2:** Treatment toxicities during RT + mEHT.

**Treatment toxicity (CTCAE v4.0)**		
**Toxicity**	**Case number (*****N*****)**	**%**
**Skin toxicity**		
Grade 0	20	60.6
Grade 1	12	36.3
Grade 2	1^*^	3.1
Grade 3	0	0
**Hepatic toxicity**		
Grade 0	31	93.8
Grade 1	1	3.1
Grade 2	0	0
Grade 3	1^**^	3.1
**Myelotoxicity**		
Grade 0	24	72.7
Grade 1	1	3.1
Grade 2	6	18.2
Grade 3	2	6.0
**Neurotoxicity**		
Grade 0	32	96.6
Grade 1	0	0
Grade 2	0	0
Grade 3	1^***^	3.1
**Nausea and vomiting**		
Grade 0	30	90.9
Grade 1	1	3.1
Grade 2	2	6.0
Grade 3	0	0
**Diarrhea**		
Grade 0	30	90.9
Grade 1	1	3.1
Grade 2	2	6.0
Grade 3	0	0
**Elevated core body temperature (after treatment)**		
Yes	6	18.2
No	27	81.8
**Fat induration**		
Yes	2	6.0
No	31	94.0

* *Autoimmune reaction: Dermatitis herpetiformis*.

** *Autoimmune reaction: Autoimmune hepatitis*.

*** *Autoimmune reaction: Myasthenia gravis*.

Among the in-field evaluable lesions treated with RT and mEHT (39 lesions in 33 patients), CR, PR, SD, and PD were observed in 6.1, 54.5, 27.3, and 12.1% of patients (Table 3). All eight breast cancer patients had ≧PR response. Somewhat surprisingly, larger tumors (>500 ml) demonstrated superior responses than smaller tumors (<500 ml) (100 vs. 48%, *p* = 0.012) (Table 3). All the patients with autoimmune toxicities had a tumor size of more than 500 ml. Because of the small sample size, multivariate analysis failed to show significant differences between response and age, tumor size, number of mEHT treatments, tumor depth, the use of ICP inhibitors, chemotherapy, and autoimmune reactions. Thirteen patients (39.4%) had a decreased N/L ratio 1 month after RT + mEHT, which includes two patients with CR, six with PR, three with SD and two with PD (Table 3). The three patients with an autoimmune abscopal effect had an elevated N/L ratio before treatment (>8) which decreased to <3.5 after treatment. The median survival time was 11.4 months (range, 2.6–16.9 months) in patients whose N/L decreased, vs. 8.9 months in patients with elevated N/L (range, 1.7–16.2 months).

**Table 3:** Response rate of the irradiated sites.

**Response**	**Metastatic/recurrent (*N* = 33)**	GTV ≥500 ml (*N* = 8)^**^	**GTV <500 ml (*N* = 25)**	Decreased N/L ratio post treatment (*N* = 13)^****^	**Increased N/L ratio post treatment (*N* = 20)**
CR	2 (6.1%)	8^***^ (100%)	12 (48%)	2	0
VGPR^*^	5 (15.2%)			3	2
PR	13 (39.4%)			3	10
SD	9 (27.3%)	0 (0%)	13 (52%)	3	6
PD	4 (12.1%)			2	2

* *VGPR, Very good partial response defined as >90% regression*.

** *p = 0.012 Fisher's exact test*.

*** *The 8 patients with large tumors included 3 patients with autoimmune toxicities including 1 CR (urothelial carcinoma); 1 VGPR (breast cancer), 1 PR (cholangiocarcinoma). Another 5 patients included 1VGPR (hepatoma) and 4 PR (1 cervix and 3 breast cancers)*.

**** *p = 0.245 Fisher's exact test*.

### Case Presentation: Autoimmune Phenomena Associated With Abscopal Tumor Response

#### Case 1

**Figure 1:**
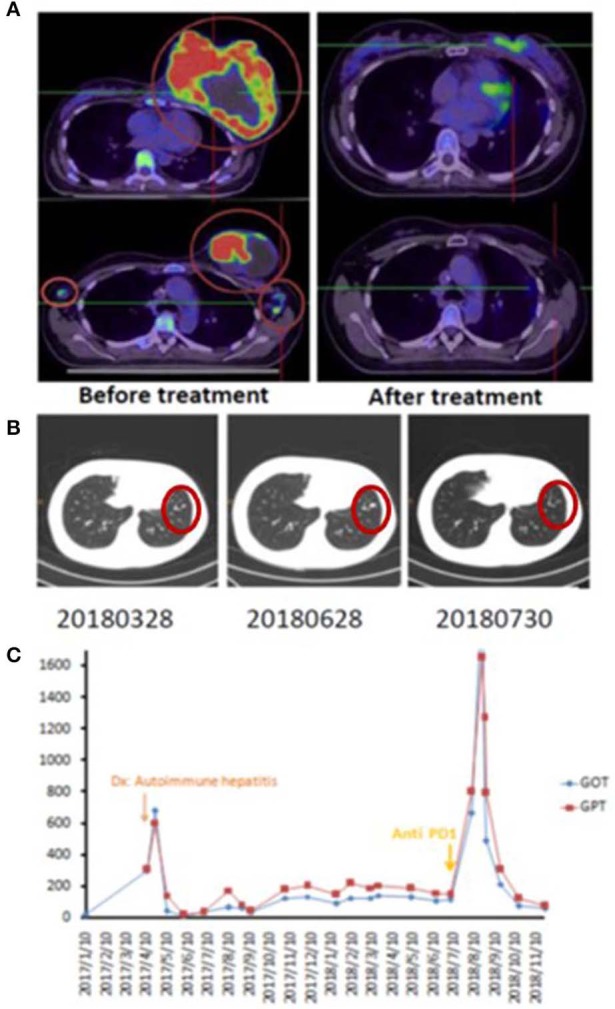
Representative patient (Case 1) with autoimmune mediated abscopal effects. **(A)** Locally advanced breast cancer with tumor abscopal effect on bilateral axillary and internal mammary lymph nodes. **(B)** Progressive lung metastatic lesions successfully salvaged with 2 cycles of low dose Opdivo®. **(C)** Flare up of autoimmune hepatitis by RT plus mEHT and ICP inhibitors.

A 42-year-old female patient presented with a left breast ulcerative fungating mass (>10 cm) with palpable bilateral axillary lymph nodes. She was diagnosed with metastatic, left breast, triple-negative invasive ductal carcinoma. She refused chemotherapy and received palliative RT consisting of 50 Gy in 25 fxs plus weekly mEHT for six treatments. Elevated serum alanine and aspartate aminotransferases (ALT and AST), alkaline phosphatase and bilirubin was identified 2 weeks after RT. Positive anti-microsomal antibody and anti-smooth muscle antibody levels assisted in making a diagnosis of autoimmune hepatitis. She was treated with prednisone (starting at 40 mg daily and tapered to 10 mg daily within 4 weeks). The primary tumor shrank rapidly to ~1 cm 1 month after treatment and a wide excision was performed 2 months later (Figure 1A). The bilateral axillary and the left internal mammary metastatic lymph nodes outside the local treatment field demonstrated dramatic and sustained regression, qualifying for our abscopal response criteria. More than 1 year later, she developed lung metastases and was treated bi-weekly with reduced-dose ICP inhibitor treatment (60 mg of Opdivo®) for two doses with a significant response (Figure 1B), resulting in a CR. Subsequently, her serum AST, ALT, and bilirubin levels increased once again, suggesting relapse of her autoimmune hepatitis, resulting in discontinuation of immunotherapy (Figure 1C). Despite this, her lung metastases demonstrated sustained remission, and she is still alive and tumor-free, >12 months after discontinuing ICP therapy.

#### Case 2

**Figure 2:**
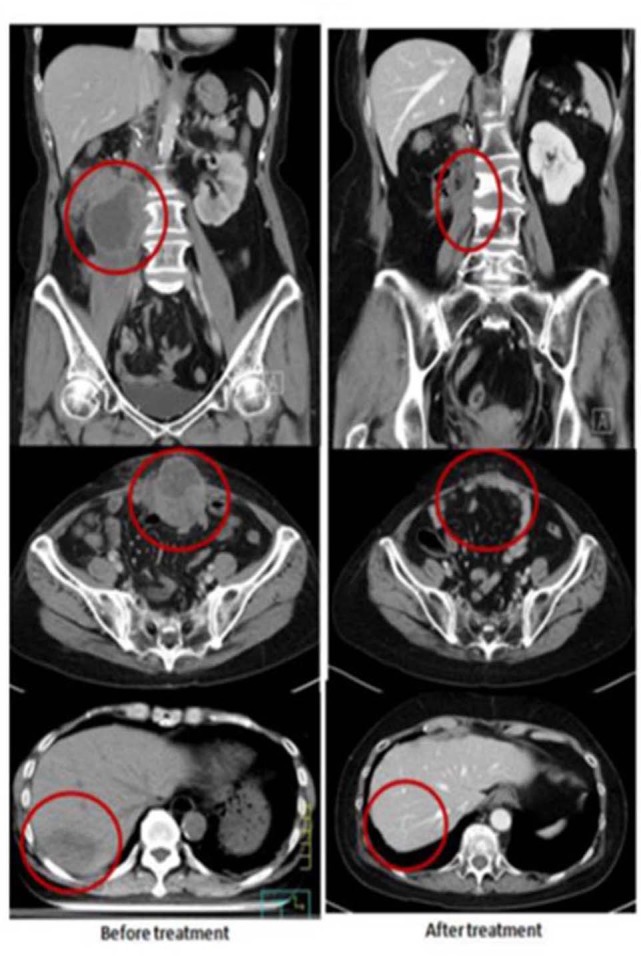
Representative patient (Case 2) with autoimmune mediated abscopal effects: metastatic urothelial carcinoma with abscopal tumor effect on liver metastases.

A 60-year-old female had right renal pelvis urothelial carcinoma diagnosed in October 2016. She underwent robotic right nephroureterectomy and bladder cuff excision, revealing a pT4N0 tumor treated with adjuvant tumor bed radiotherapy to 48 Gy in 24 fxs (completed in January 2017). In May 2017, she presented with a rapidly growing, painful, palpable abdominal mass. An abdominal CT scan showed multiple intra-abdominal masses and a right retroperitoneal mass attached to the right psoas muscle. The largest tumor was 4.5 cm. There was also a separate lower anterior abdominal wall mass and a liver segment 7 metastases. She received a second course of palliative RT targeting the symptomatic and dominant right lower quadrant mass and the lower abdominal wall mass, both treated to 40 Gy in 20 fxs, along with five weekly mEHT treatments. Concomitant carboplatin at 300 mg and gemcitabine at 600 mg were given for only two cycles and discontinued after pancytopenia developed. The abdominal pain resolved quickly, and she developed a mild fever with elevated CRP and pancytopenia in the 3rd week of treatment. A generalized itchy skin rash developed over the trunk in the 4th week of treatment. She was diagnosed with dermatitis herpetiformis and macrocytic anemia with positive anti-parietal cell antibody. The skin lesions were controlled with low-dose prednisolone (10 mg, once daily). A CT scan in August 2017 showed CR at the irradiated sites. Unexpectedly, an abscopal effect of the hepatic metastases was also identified (Figure 2) which was unlikely to be from the systemic effect of only two cycles of low doses of carboplatin and gemcitabine. No further treatment was administered. A recent follow-up CT scan in May 2019, 2 years after palliative RT, showed persistent CR of all disease sites.

#### Case 3

**Figure 3:**
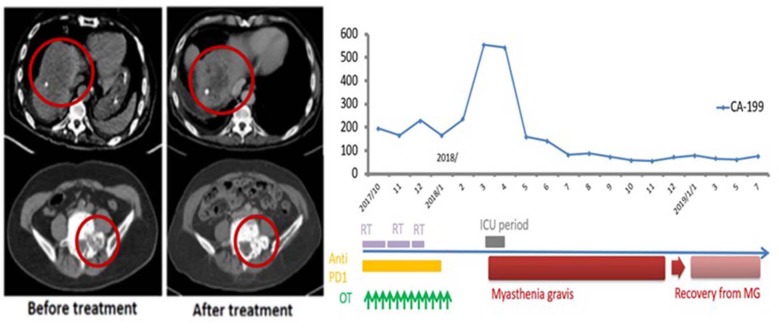
Representative patient (Case 3) with autoimmune mediated abscopal effects: metastatic cholangiocarcinoma with abscopal tumor effect on L5 bony metastases and change of CA-199 level.

This 69-year-old male patient had a biopsy-proven cholangiocarcinoma with multiple metastases diagnosed in August 2017. He began treatment with immunotherapy (Yervoy® at 50 mg for one dose only and Opdivo® at 60 mg every 2 weeks) for eight cycles, RT (45 Gy in 15 fxs to the liver, 30 Gy in 10 fxs to the scapula, L1 spine, and right pelvic bone), and weekly mEHT for 12 sessions starting from September 2017. In December 2017, he suffered from progressive muscle weakness with mild ptosis, lethargy, and difficulty in swallowing. He developed an aspiration pneumonia, requiring intubation and supportive management in the neurology intensive care unit. A positive acetylcholine receptor (AchR) antibody with electromyogram findings confirmed a new diagnosis of myasthenia gravis (MG). The patient gradually recovered after receiving plasmapheresis, steroids, and antibiotics. He did not receive any further anti-neoplastic therapy and was maintained on prednisolone, 5 mg once daily, for the subsequent 10 months. Follow-up imaging showed good PR at irradiated sites with measurable PR of the unirradiated L5 spine metastases. The CA 19-9 level peaked to 555 U/ml in 2018/3 and gradually dropped to 76.7 U/ml in 2019/6 (Figure 3) and he remained asymptomatic without any systemic treatment.

## Discussion

Although our patients were heterogeneous in terms of histology, lesion numbers, and prior treatments, in general, they represented a relatively common pool of patients referred for palliative radiotherapy, i.e., relatively large, symptomatic disease, either heavily pretreated or having declined during other therapies. In that context, the 60.6% overall response rates of the locally treated (RT plus mEHT) lesions may suggest a synergistic or radiosensitizing effect. Unexpectedly, tumors larger than 500 ml, had an even better response rate. Intriguingly, three cases of autoimmunity occurred after treatment, which was associated with abscopal tumor response.

What possible mechanisms could be at play here? The combination of systemic autoimmune effects and tumor abscopal effects provoked by combined mEHT and RT from local treatment leads us to speculate the possibility of the clonal expansion of a subset of T cells targeting both tumor antigens and shared normal tissue epitopes. These patients required steroids for managing their autoimmune reactions, without loss of tumor control. mEHT induces tumor cytotoxicity through a combination of localized thermal effects, and the temperature independent signal-excitation effect for DAMP release (12, 30). HSP-associated DAMP could facilitate immunogenicity, especially in the context of concomitant RT and possible combination with immune checkpoint inhibitors. The addition of ICP inhibitors after concomitant chemoradiotherapy (CCRT) in stage III lung cancer patients, improves both progression-free and overall survival rates relative to any other consolidative approach, suggesting the possibility that localized therapy creates a milieu for ICPs to have more durable effects (31, 32).

Are the local and abscopal responses reported herein, especially their depth and durability, expected and routine? Patients in this study were generally at such an advanced stage of their disease, that first, the expected response rates would be rather low, and second, durability would be very uncommon. This leads us to hypothesize that our clinical observations would require the development of unleashed anti-tumor autoimmunity, possibly from combinatorial mEHT and RT. In the three cases with autoimmune toxicity, Case 1 was treated with RT and mEHT only; Case 2 received only two cycles of reduced dose concomitant chemotherapy and Case 3 had immunotherapy with RT. It would be very unlikely that the abscopal liver metastasis response in Case 2 was a chemotherapy effect. Whether the remote bony metastases response in Case 3 qualified as a pure “abscopal effect” is debatable. Nevertheless, the autoimmune reactions in the three cases after local treatment were quite clear. Immune response through *in-situ* vaccination might be amplified by the addition of ICP inhibitors as the third case described or might yield a deeper response as the second case described. Gauci et el. recently reported that in order to prolong survival, a CR or PR within 3 months after treatment was mandatory with anti-PD-(L)1 monotherapy for multiple cancer types (33).

Larger tumors had better responses to combined RT and mEHT treatment, which is counterintuitive. Explanations for this include the possibility that large tumors, especially those near the body surface under the electrode, absorb more energy from the RF current (34). For example, in an *in vivo* experiment, the use of a large 20 mm diameter electrode to deliver mEHT to 8 mm diameter size murine tumors resulted in impressive apoptosis, necrosis, and extracellular damage-associated molecular secretion patterns (12, 13), presumably because the entirety of the tumor was able to absorb energy effectively. Similarly, all eight breast cancers responded to this treatment. The radiation fractional dose used in our series is classic and typical for palliative radiotherapy, but atypical as far as several preclinical combinatorial immune checkpoint-radiation experiments recommend (for example 5–9 Gy per fraction, 3–5 fractions). Despite the use of lower fractional doses, the responses observed herein are robust. This could reflect the combinatorial use of mEHT. However, it is also worth considering that other clinical reports, such as the one by Chandra et al., demonstrated that radiation fraction size <3 Gy was the only parameter identified to be associated with favorable index lesion response in a cohort of melanoma patients treated with immune checkpoint inhibitors and radiotherapy (20).

As the use of immunotherapy becomes more popular, irAE is emerging as an issue (35). irAEs from ICP inhibitors are generally regarded as a “toxicity,” however, a number of reports are beginning to appear in the literature claiming that patients with higher irAEs may have a higher response rate (36, 37). In addition, patients with irAE have longer treatment durations and more time to develop autoimmune toxicities (35). Clearly, immunotoxicity and autoimmunity is a balancing act. In our study, three out of 33 patients (9.1%) had induced autoimmune reactions from RT + mEHT. They all had a profound abscopal effect (>90% shrinkage of non-irradiated tumor, lasting for more than 12 months) without any substantial systemic targeted, cytotoxic, or ICP inhibitor therapy when autoimmune toxicities were noted. Most abscopal effects reported in the literature are neither “deep” (i.e., >90% tumor reduction), nor durable. For example, in the series by Golden et al. (23), only two of 41 patients (4.9%) had a dramatic abscopal response according to the criteria of >90% tumor reduction. Therefore, we argue that the effective immunity may be coupled to autoimmunity.

This case series of combining mEHT and RT for palliative purposes demonstrated unexpected autoimmune toxicities along with dramatic and sustained tumor regression. Despite being interesting and inspiring, these results must be interpreted with great caution and at best provide initial observations for hypothesis-generation, as there are considerable limitations given the retrospective, single institution analysis, with limited patient numbers, and considerable heterogeneity. An official prospective trial combining immune check point inhibitors with RT and mEHT will be launched.

## Data Availability Statement

All datasets generated for this study are included in the article/supplementary material.

## Ethics Statement

The studies involving human participants were reviewed and approved by Institute Review Board of Shin Kong Wu Ho Su Memorial Hospital. Written informed consent for participation was not required for this study in accordance with the national legislation and the institutional requirements.

## Author Contributions

M-SC, MM, and K-HC: conception and design and writing review of the manuscript. K-LY, H-CL, Y-SW, H-LK, Y-CL, and K-HC: development of methodology. M-SC: acquisition of data and analysis and interpretation of data (e.g., statistical analysis, biostatistics, computational analysis). K-HC: study supervision. All authors read and approved the final manuscript.

### Conflict of Interest

The authors declare that the research was conducted in the absence of any commercial or financial relationships that could be construed as a potential conflict of interest.
